# Meaningful approaches to content selection and ways of working: Norwegian instrumental music teachers’ experiences

**DOI:** 10.3389/fpsyg.2023.1105572

**Published:** 2023-02-03

**Authors:** Anne Jordhus-Lier, Sidsel Karlsen, Siw Graabræk Nielsen

**Affiliations:** ^1^Department of Arts and Cultural Studies, Inland Norway University of Applied Sciences, Elverum, Norway; ^2^Norwegian Academy of Music, Oslo, Norway

**Keywords:** musical upbringing and schooling, schools of music and arts, instrumental teaching, inclusion, exclusion, sociology of music education, music Didaktik, music teaching

## Abstract

The Norwegian municipal schools of music and arts are publicly funded institutions which offer extra-curricular activities for children and adolescents in music and other art forms. The system is designed to ideally reach all children, but while each municipality is legally responsible for providing school of music and arts education for its inhabitants, the law does not state anything about teaching content or ways of working. Consequently, this is up to the teachers to decide. What is taught in these schools is, however, relevant to whether children feel included or excluded. This means that music teachers’ beliefs and actions are among the factors that influence who will feel welcomed and who will feel alienated. On this basis, in this article we explore music teachers’ approach to content-related decision-making processes by asking about their meaningful approaches to selecting content and ways of working within instrumental music teaching. To answer that question, we discuss what kinds of teaching content the teachers choose in general, as well as for beginner and advanced students, the reasons they express for selecting content, which, if any, music they find not to be suitable as teaching content, and how they work with the selected repertoire. We draw on empirical data from a survey among 151 music teachers and an interview study with 11 music teachers. Discussing results from these sources of data in relation to the Nordic and German music Didaktik theories, enables us to address meaningful approaches to selecting content and ways of working. From the analysis, we draw conclusions about what the music teachers experience as meaningful approaches. These can be summarized as (a) the centrality of the students in the process of selecting content; (b) genre versatility, meaning that students should be exposed to a broad range of musical genres and styles; and (c) that students are exposed to the “classical repertoire,” or the standard repertoire within a genre or tradition. In general, what seems to be meaningful for the teachers is working “close to the student’s wishes and preferences,” but in ways that relate to a variety of Didaktik principles.

## 1. Introduction

The Norwegian municipal schools of music and arts are publicly funded institutions which offer extra-curricular activities for children and adolescents in music and other art forms. The system is designed to ideally reach all children as each municipality is legally responsible for providing school of music and arts education for its inhabitants (Norwegian Education Act, 1998, pp. 13–16). The law does not state anything, however, about what forms of teaching content are desired or which ways of working with the musical material should be preferred. Although a detailed and quite recently updated curriculum framework exists (Norwegian Council for Schools of Music and Performing Arts, 2016), this does not provide specific guidelines for repertoire or how to go about teaching it. The curriculum framework is only advisory, hence, the schools are not obliged to follow it. Consequently, it is to a great extent up to the teachers to decide on the content and on ways of working. Since such deliberations are left up to the teacher, we took this as a point of departure for exploring which approaches to content selection and ways of working were found meaningful by instrumental music teachers working within Norwegian schools of music and arts.

The schools of music and arts are not part of the compulsory school system, rather they are music and arts centres offering voluntary arts courses. They are publicly financed, but students pay a fee set by the various municipalities. The schools have no entrance examinations. If there are not enough available places, applicants are put on waiting lists. From the very beginning of the Norwegian music and art school system’s existence, popular music and folk music have been included, if not with the same self-evidence as art music/classical music. Previous research reports quite a strong classical music hegemony that has been visible, among other things, in all curriculum frameworks, including the most recent one (Ellefsen and Karlsen, 2020; Karlsen and Nielsen, 2021). However, when exploring empirically which musics are currently played and offered, we find that popular music and classical music seem to occupy almost equal space and status, both when it comes to which instruments and ensembles are taught and facilitated on the national level (Jordhus-Lier et al., 2021) and which forms of repertoire the students are expected and encouraged to play (Nielsen et al., 2022). In addition to these two most frequently occurring broader genres, certain music and art schools also teach Norwegian folk music, Sami music, and the music of some immigrant minority groups, although the distribution of such genres is geographically limited and determined (Jordhus-Lier et al., 2021).

Previous research also gives indications as to what may influence the selection of repertoire in Norwegian music and arts schools. Jordhus-Lier (2018) has shown that music teachers’ own genre versatility may influence teaching content to quite a large degree. This form of versatility is also one of the sought-after competences when hiring teachers, and increasingly so. We also know that, in general, the teachers’ influence is strong when musical repertoire is selected for teaching (Nielsen et al., 2022), and even more so when teaching art music/classical music than when teaching popular music repertoire. To a certain degree, instructional textbooks and other educational material also seem to set the premises for what is taught (West and Rostvall, 2001; Nielsen et al., 2022), perhaps especially at the beginner level (Blix, 2018).

The teachers’ central role in selecting content and ways of working may partly be understood through a quotation from Holmberg (2010), in which she claims that music and arts school teachers in Sweden see themselves as “defenders of their own practice, the last lifeline of highbrow culture in a stormy sea of cultural relativism” (p. 196). That music and arts schools are seen by many as a site for the dissemination of highbrow culture is supported in Norwegian research as well (see, e.g., Gustavsen and Hjelmbrekke, 2009; Berge et al., 2019). This, combined with the fact that even a low-priced fee can make attendance difficult for children and youth from socio-economically disadvantaged backgrounds, plus the observation that the schools are an unknown phenomenon to a large group of the population (Bjørnsen, 2012)—to the extent that they are described as a well-kept secret (Berge et al., 2019)—make the music and arts schools arenas for quite effective cultural inclusion and exclusion, often along lines of division related to social class. Both Swedish and Norwegian research emphasize that music and arts schools are playgrounds predominantly designated and accessible to middleclass users (Jeppsson and Lindgren, 2018; Berge et al., 2019; for more information about class structure in Norway, see Hansen et al., 2009). Indeed, Finnish researchers have recently described the opportunity gap that results from the seemingly free choice of extra-curricular music activities in Finland as a form of hidden elitism (Väkevä et al., 2022).

Although the social dynamics related to extra-curricular schools of music and arts can be seen as connected to macro-scale social systems, and thus can be analyzed on the systems level of society, we believe that the everyday workings of such dynamics should also be given attention, and that they are perhaps most efficiently explored through teachers’ approaches to content selection and ways of working with the musical repertoire. Here, we work from a Bourdieusian-inspired theoretical understanding which implies that we see music as the art form that most “clearly affirms one’s ‘class’” (Bourdieu, 1984, p. 18), and whose related patterns of taste, use, consumption, and (re) production thus also work to divide and classify people, and also that the micro and macro levels of society mutually constitute each other through “[t] he practical sense” (Bourdieu, 2000, p. 139) and embodiment of structure that is provided by habitus. As such, in this specific practice, the approaches to selecting content and ways of working qualified as necessary and meaningful to the teachers will be heavily imbued with their embodied practical sense (Bourdieu, 1990). Consequently, instrumental music teachers’ beliefs and actions are, on the everyday micro level of society, among the factors that will most strongly influence who will feel welcomed and included in the music and arts school system, and who will feel alienated and excluded. On this ground, in this article we explore music teachers’ approach to content-related decision-making processes by asking:

What are meaningful approaches to selecting content and ways of working within instrumental music teaching?

- What kinds of teaching content do the teachers choose in general, as well as for beginner and advanced students, respectively?
- What reasons do the teachers express for selecting content?
- Which, if any, musical styles and genres do the teachers find to be unsuitable as teaching content?
- How do the teachers work with the selected repertoire?

This study is part of a larger research project investigating the social dynamics of musical upbringing and schooling in Norway (DYNAMUS, n.d.), where the system of extra-curricular schools of music and arts was used to exemplify one vital arena (see Jordhus-Lier et al., 2021; Karlsen and Nielsen, 2021; Nielsen et al., 2022).

## 2. Theoretical perspectives on music Didaktik

When examining how music teachers select and decide how to work with teaching content, we find it fruitful to draw on some central elements from the Nordic and German Didaktik and music Didaktik traditions. Thus, we discuss our findings in relation to Klafki’s (2006, 2011) Bildung and Didaktik theories, as well as Nielsen’s (1998) five central activities. In addition, we also include Dyndahl and Ellefsen’s (2009) discussion on how the field of cultural studies might inform music education scholars’ understanding of the complexities involved in Didaktik-related choices.

The word Didaktik derives from a Greek word meaning to teach/to be taught, and refers to the art of teaching (Johansen, 2007; Nielsen, 2007). Didaktik as a scientific discipline is closely linked to the tradition of German humanities of the last 200 years, including the concept of Bildung (Nielsen, 2007). Within the Nordic music educational field, Nielsen (1998) has been central in further developing these traditions. Music Didaktik as a pedagogical tradition concerns the art of teaching music and deals with the rationale for upbringing and music education; that is, *why* students should learn music and *what* they should learn (Nielsen, 2007; Johansen, 2017). It includes all the decisions that teachers have to make, as well as the rationale behind such decisions. Didaktik connects theoretical and practical considerations, and Nielsen claims that

a widespread understanding of the object of study of Didaktik can therefore, conditioned by the specific Didaktik concept, be summarized in the following definition: Didaktik deals with the theory and science as well as the planning and decision-making of the content, aim and rationale of teaching/learning (Nielsen, 2007, p. 267).

A narrow understanding of Didaktik is common within the Danish music Didaktik tradition represented by Nielsen (1998), focusing on the aims and content of the teaching. In Norway, however, a broad understanding, in which the methods or methodology are included, is most common (Nielsen, 2007; Hanken and Johansen, 2021). This is also reflected in the present study, where we focus on which content the teachers select, their reasons for selecting the content, and also how they work with the selected content. Klafki originally focused primarily on content-and curriculum-related issues, but later used the term Didaktik for both the dimensions of objectives and content and the dimension of methods (Klafki, 2006). He asserted, however, that method planning can only take place after Didaktik analysis because methodological steps are “governed by practical considerations, whereas the order of Didaktik reflection follows theoretical-systematic norms” (2006, p. 130). In other words, the “pedagogical significance and structure of which have been established by Didaktik analysis” must be the base from which one can find the ways that lead to “the fruitful encounter between the children and the content” (Klafki, 2006, p. 129). Catering to this encounter constitutes the purpose of instructional preparation, according to Klafki (2006, 2011). Within Klafki’s ideas of categorical Bildung, exemplary teaching, and critical-constructive Didaktik, we can find answers to how one can work toward achieving such goals.

To develop his theories, Klafki analyzed the history and development of the concept of Bildung. He found two main understandings of the concept, namely material and formal Bildung theories (Nielsen, 1998; Klafki, 2011; Straum, 2018). Within material Bildung, the acquisition of the content is the goal, either as “objective knowledge,” in which the students are introduced to the society’s knowledge, ethics, and so forth (objectivism), or as the classical content within a culture, defined by what dominates or has achieved status (classical Bildung) (Nielsen, 1998; Klafki, 2011, p. 15; Straum, 2018; Hanken and Johansen, 2021). Within the tradition of formal Bildung, the student is the central element, and the focus is either on developing the student’s inherent abilities (functional Bildung) or on the process of learning methods to achieve strategies to master life (method-based Bildung) (Nielsen, 1998; Klafki, 2011, p. 15; Straum, 2018; Hanken and Johansen, 2021). Hanken and Johansen (2021) see these different Bildung theories in connection with upbringing toward music (material Bildung) and upbringing through music (formal Bildung). Nielsen (1998) asserted, however, that it is seldom a matter of upbringing toward or through music, but rather a combination of both. This is in line with Klafki, who argued that neither material nor formal Bildung theories can stand on their own; when focusing on the content one needs to see it in relation to the student and the society, and focusing on methods without any relation to content is neither productive nor even possible (Straum, 2018). Instead, Klafki argued in favor of combining these theories, in which Bildung happens in a dialog between the teaching content and the student, where the object and the subject are inextricably linked in a dialectical unity (Nielsen, 2007; Klafki, 2011; Straum, 2018). This is often referred to as a “double unlocking”; the content is “unlocked” for the student and the student is “unlocked” to the content and its relation to the surroundings (Klafki, 2011, p. 17; Straum, 2018). This Bildung theory is what Klafki named categorical Bildung. A central element within categorical Bildung is exemplary teaching, which, in short, refers to the idea of enlightening abstract, general, and fundamental principles by focusing on a few concrete (and good) examples, which are, or can be, connected to the students’ world outside the school (Johansen, 2007; Klafki, 2011; Straum, 2018). An element of critique is at the core of Klafki’s later Didaktik theory which he denotes critical-constructive Didaktik (Klafki, 2011), where students’ self-determination as well as exemplary teaching are central. The main goals for teaching within critical-constructive Didaktik are self-determination, co-determination, and solidarity, which can be achieved through exemplary teaching (Klafki, 2011).

Within the art of teaching, Didaktik analysis is crucial, according to Klafki (2006), and the focus should be on what is to be taught: the content. The teacher’s task is thus “to elucidate which aspects of the content contribute to Bildung, to explore what it contains that can or should comprise education, Bildung” (Klafki, 2006, p. 117). Klafki (2006, p. 118) also emphasized the importance of the teacher adopting or representing two positions: (a) the “lay-person” the student will become; for instance, “the democratic citizen,” and (b) the young person the student is, with his or her capacity for understanding where the teacher “explore [s] them for their deeper educational potential.” Within the first position, Klafki (2006, p. 118) emphasized the relevance of teachers to be “willing to be moved by the subject matter during preparation.” Connected to this is the importance of selecting content that is relevant to the students. This is important in at least two ways: (a) as a connection to the society and to activities outside the school (where the students are now), and (b) as significant for the students’ future (Klafki, 2006).

The connection between content and activity is illustrated by Nielsen (1998), who exemplified how a song as content (the song the students should learn) could be selected based on either the song itself (as from a specific period, from a certain country, etc.), or on the activity of singing. Nielsen (1998, 2007) also described five subject-related forms of activity which should be “understood as a way the student can engage (be ‘actively’ involved with) the medium and the object area in question” (2007, p. 273). The five central activities are *reproduction*, *production*, *perception*, *interpretation*, and *reflection*, where the first four imply being in direct contact with the music and the fifth means reflecting about music (Nielsen, 1998, 2007). These central activities are intertwined, as elements from other activities will be present, although one form of activity is central. In the central activity, *reproduction*, to perform and reproduce existing music, either by singing, playing an instrument, or leading others, is central. This implies the need for a representation of the music; the music needs to be written down or recorded, or handed over orally. A central element within this activity is the implication of the idea of an existing musical “piece” with an identity that is maintained (or at least attempted to be maintained) from one performance to another (Nielsen, 1998, 2007). The development of musical skills is fundamentally connected to playing existing music, according to Nielsen (1998), and this activity has historically been central to content selection in music education.

*Production* is connected to creating, composing, and improvising music, and *perception* concerns listening to music, representing a receptive relation to music (Nielsen, 1998, 2007). Nielsen (1998) underlined, however, that the activity of perception cannot be understood in isolation because all musical activities imply listening at some point. With *interpretation*, Nielsen (1998) referred to the activity of analyzing and interpreting music and expressing its understanding and interpretation in a non-musical medium, which is to say an analytical and hermeneutic form of interpretation. The last central activity, *reflection,* concerns thinking about music, where considering, investigating, and seeing music in historical, sociological, and psychological perspectives are central (Nielsen, 1998, 2007).

Another approach to music Didaktik can be found in Dyndahl and Ellefsen’s (2009) exploration of how perspectives from cultural studies can inform music education scholarship, and in particular the subfields interested in the Didaktik-related “aspects of teaching and learning music” (p. 9). These contributors lead a discussion mainly on didactology as a meta-level, and demonstrate how the identity of the subject of music itself is “complex, contingent and culturally contextual” (p. 9). However, they also put the students’ identities at the forefront when discussing how various ways of facilitating music education might offer different subject positions:

How does the school subject music work as a field of education where pupils and students negotiate, renegotiate and identify with narratives of themselves as male/female, straight/queer, white/black, native/foreign, local/cosmopolitan, young/grown-up practitioners and participants in musical activities and communities … and for that matter experience a sense of belonging to high/low social class and/or culture as well? (p. 16)

In our opinion, this multifaceted understanding of the experiential and existential negotiations taking place within the frames of the music subject is not only valid for the context of (compulsory) schooling. It is also highly relevant when considering how content selection by instrumental music teachers may, or may not, interplay and create meaningful connections with their students’ various identities.

## 3. Materials and methods

The present study has a multi-method approach (Creswell and Plano Clark, 2007), combining data from interviews and a survey questionnaire, both conducted among music teachers in schools of music and arts. Multi-method studies are studies in which multiple types of qualitative or quantitative data are collected, and are distinguished from mixed methods studies collecting *both* qualitative and quantitative data (Creswell and Plano Clark, 2007, p. 273). In the present study, we combine the survey’s open-ended questions with interview data. We thus let two types of qualitative data inform each other: the survey adds rigor to our data from the interview study, and the interviews add depth.

### 3.1. The questionnaire and the interviews

The questionnaire was distributed electronically among 902 music teachers at 70 schools of music and arts between October 2019 and May 2020. The schools were selected using a quota sampling strategy (De Vaus, 2013) based on geography (18 counties in 2019)^1^ and municipal size.^2^ We received 151 responses, with good geographical and size-related dispersion: the music teachers were from all 18 counties; about half of them were from medium-sized municipalities, about a quarter from small municipalities and a quarter from large municipalities (see also Nielsen et al., 2022). Encouraged to choose two music examples from their most recent teaching day, the teachers described, among other things, these examples in terms of title, composer or artist, and how they worked with them as teaching content. In addition, they were asked to share an example of other pieces of music they preferred to use in their instrumental or vocal teaching, and which they thought worked well in this context. They were also asked to describe how they usually worked with this music. All these questions were open-ended, and in this article, we mostly draw on the data from the teachers’ answers on how they worked with the music examples provided.

In the semi-structured interview study (Brinkmann and Kvale, 2015), 11 music teachers at five different schools of music and arts participated. The teachers belonged to five strategically sampled schools situated in different parts of Norway. The selection of these schools was based on results of a study by Jordhus-Lier et al. (2021), which mapped Norwegian schools of music and arts’ offerings in terms of musical genres and related instruments and ensembles. We were thus able to choose our five schools according to variation in geographical location, size of municipality and musical instruments and genres. The 11 teachers were selected to represent the schools’ various profiles and variations in instruments and musical styles taught. The interview guide consisted of four topics: (a) the teachers’ background; (b) their choice of teaching content; (c) their views on the school of music and arts; and (d) their experiences with involving parents and keeping in contact with their students. In this article, we focus on what the teachers told us about their choice of repertoire for beginners and advanced students, respectively, focusing both on repertoire choices and the reasons behind them. We also asked the teachers if they found some musical styles or genres not suitable as teaching content for these groups of students.

### 3.2. Participants

The survey teachers and the interviewees had musical backgrounds from diverse musical genres, such as folk music, art music/classical music, popular music, wind band, and other genres.^3^ The survey teachers taught a wide range of instruments (for detailed information, see Nielsen et al., 2022), while the interviewees taught piano, singing, string instruments, popular music instruments, folk music instruments, and wind band instruments. Of the survey respondents, 142 teachers (94%) had formal music education, and 127 (84.1%) had formal pedagogical qualifications (Nielsen et al., 2022). Of the interviewees, all but one had formal education in music, and 9 of 11 were educated as music teachers with formal pedagogical training. Three of the interviewees were educated as school music teachers and also taught music (or other subjects) at primary and lower secondary schools as part of their jobs in the municipalities. The interviewees also differed with regard to their level of education in music. One teacher had completed two out of three years as part of a bachelor’s degree in music, while others held a bachelor’s, master’s or doctorate degree in music. The interviewed teachers had been working at their respective schools of music and the arts for between two and almost 30 years. Most of their students were between year 6–15, which is the school of music and arts’ main target group. The least experienced teacher taught folk music instruments, while the two most experienced teachers taught string instruments, working mainly within the classical music genre. Only two of the teachers were not expected to include ensembles as part of their work, while others taught and conducted wind bands, string ensembles, popular music bands, and choirs within the schools. All but one teacher were active performing musicians, playing solo concerts, participating in Kappleik,^4^ conducting or playing in amateur ensembles (choirs, wind bands), or playing in professional ensembles (symphony orchestra, popular music band), composing and recording records or being studio musicians.

### 3.3. Analysis

All the data material was analyzed qualitatively, using thematic analysis, as it “can be applied *across* a range of theoretical and epistemological approaches” (Braun and Clarke, 2006, p. 5). The interviews were first transcribed and then anonymized. Then, the data material, both the interviews and the open-ended questions from the survey, were coded separately using NVivo. The codes were built from the material during the coding process, according to the principle of open coding (Kvale and Brinkmann, 2009; Tjora, 2018), in combination with organizing the codes into categories connected to the research questions. While coding was done by only one researcher, the categories constructed were discussed with the other two researchers during the process of analysis so as to strengthen the reliability of the findings. The original language of the data is Norwegian. The coding was done in Norwegian, and later translated into English by the researchers.

We draw on both the interviews and the survey in order to answer our research questions. When answering *what kinds of teaching content the teachers choose in general, as well as for beginner and advanced students*, we use data material from both. To give answers to *which reasons the teachers express for selecting content*, as well as to *which, if any, musical styles and genres the teachers find unsuitable as teaching content*, the interviews are the data source. Finally, we use data from both the survey and the interviews to provide answers to *how the teachers work with the selected repertoire*. These answers will be discussed in relation to previous research and music Didaktik traditions (Nielsen, 1998, 2007; Klafki, 2006, 2011; Dyndahl and Ellefsen, 2009) in the discussion chapter, where we will answer our main research question: namely, *what are meaningful approaches to selecting content and ways of working within instrumental music teaching?*

### 3.4. Ethical consideration

Both the survey and the interview study were submitted for ethical evaluation to the Data Protection Service of the Norwegian Agency for Shared Services in Education and Research, and subsequently approved. All informants were given ample information about the research prior to the interviews, and they also signed an agreement giving their informed consent. The interviewees and their schools have been given fictitious names. The schools are named after where in Norway they are located: North, Middle (in the middle of Norway), West, South, and East, and the teachers are referred to by their school and a number.

### 3.5. Limitations

Our analyzes are based on data from a relative small sample of music teachers in the Norwegian schools of music and the arts, and thus, the results are only to a limited degree generalizable to how other music teachers work. Another limitation is that part of our analyzes is based on fill in-questions in a questionnaire that provided less detail on repertoire and ways of working than the interviews did. Nevertheless, the detailed analysis and theorizations may prove relevant to other music teachers working in the schools across musical genres as well as for other studies on decisions music teachers make in their classrooms.

## 4. Results

### 4.1. The chosen teaching content

The analysis of the interviews with the music teachers provided us with answers about (a) what kind of teaching content the teachers choose in general, as well as for beginner and advanced students, (b) which music, if any, they find not suitable as teaching content, and (c) reasons they might have for selecting the content. First, we asked the teachers to give examples of teaching material they used in general. Most central here was that the teachers not only allowed their students to come up with suggestions of songs and pieces to play, but that they encouraged them to do so. This view was expressed by teachers within the classical genre as well as teachers within the genres of popular music and Norwegian folk music. Some of the teachers also talked about how they wanted their students to express their musical preferences:

If the students wish to play something, I believe one ought to stretch far to make it happen (North, teacher 2).

After one year, I am concerned about the students having their own musical preferences. … The most fun is when the students come up with suggestions of what to work on. But they need help acquiring it (Middle, teacher 1).

I usually ask them [the students] if they have a song they like. We try to play things they actually listen to, which they could have sung in school. … I make some easy versions of the riffs or the chorus so that they can play it. I think that is a way to become interested in the instrument (South, teacher 2).

One of the teachers gave a reason for asking students to bring in songs they want to play: namely, that playing these songs is a way into an interest for the instrument. Some of the teachers also emphasized the importance of students becoming independent, and to be able to choose music to play could be seen as part of that independence.

When asked to give examples of teaching content, several teachers mentioned folk music, especially from Norway, Sweden, and Ireland. One of the Norwegian folk music teachers also used Irish folk music, while some of the classically trained teachers often used folk music from Norway and other countries. One of them explained that using folk music as teaching content leads to the students establishing a different relationship with their body and their way of playing music; they “become more relaxed and less stiff.” Examples that only a few of the teachers mentioned are music from films, ear training exercises, and “local things.” Some emphasized that they advised their students to listen to different things, while others often used music they are used to from when they were students themselves. Some of the teachers also expressed that it is challenging to find repertoires that the students are interested in. This shows the importance of students liking the music when teachers are selecting content. Another element visible through the analysis is the idea of selecting repertoire based on what the student “needs,” which relates to the student’s progression and development.

When asked about the selection of teaching content for *beginners*, the teachers’ most prominent answers were beginner books/tutor books and “songs the students have heard before.” Some of the teachers also highlighted ways of working with beginners when asked about teaching content; namely, that they focus on the students getting to know the instrument and playing by ear. Famous children’s songs were also emphasized. Regarding the *advanced* students, the teachers accentuated the importance of the students themselves choosing the repertoire. They also emphasized group/ensemble playing as important for advanced students. Some of the teachers chose mostly various classical repertoires and etudes/studies as teaching content for advanced students.

We asked the teachers whether they found some musical styles and genres *not suitable* as teaching content. Only one of the teachers mentioned a style/genre, namely “death metal singing,” which she “stays away from because it may not be very suitable for lessons.” A string teacher highlighted the difficulties of playing some of the pop songs that her younger students sometimes bring into class, because “the melody only uses one note” and they are “only rhythmical.” She found these songs meaningless and “musically unattractive.” One teacher emphasized that she avoids songs with “not suitable lyrics,” even though she teaches an instrument and not vocal. Other than these examples, the teachers asserted that there is no music that is not suitable as teaching content, or that what is not suitable for the specific instrument is what is not suitable as teaching content. Some of the teachers also admitted that music they do not use as teaching content is connected to the limitations of their own competence.

When giving reasons for selecting specific content, about half of the teachers expressed variation and genre breadth as goals, and thus provided their reason. One example of this is a teacher who was teaching her piano students to play both chords and classical sheet music:

I believe that most of the students think it is okay to play a lot of different things, getting used to playing chords, using your ear and so on. In addition, they should be trained in technique. Then it works best to use more traditional piano music that leans towards the classical. I find variation to be important (Middle, teacher 2).

Other reasons mentioned by some of the teachers are goals such as the students becoming independent, broadening the students’ horizons (which is connected to variation and genre breadth), experiencing the joy of music, forming a relationship with the music, feeling mastering, and progression.

The survey participants, 151 teachers, were asked to provide two examples of music used as teaching content during their most recent day of teaching. From this, we found that *popular music* and *art music/classical music* predominated and were almost equally represented. Less-frequently mentioned examples included music from *films and TV series*, *educational material*, *Christmas music*, *folk music*, and *children*’*s music*. It is also notable that only one of the teachers reported to have used the *student’s own composition* as teaching content. In addition to describing the music the teachers used on their last day of teaching, we also asked them to list examples of music they *preferred* as teaching content. These examples show, maybe to a larger degree, which teaching content they find to be meaningful. From these answers, we found *popular music* and *educational material* to be most favored, followed by *art music/classical music* and music from *film/TV series/game tunes*. Seeing these results together, we find that *popular music* is both most used and preferred as teaching content, and that the teachers expressed a greater preference for *educational material* than what was revealed in their accounts of the previous day of teaching, while *art music/classical music* was less favored (see also Nielsen et al., 2022).

### 4.2. Ways of working

To answer how teachers work with their selected repertoire, we lean on both the survey and the interviews as data material. In the interview material, the teachers focused on ensemble playing, but also that they adapted how they work with the repertoire to the various students. They also expressed expectations they have toward the students between the lessons. The most prominent expectation is that the students should practice. Quite a few of the teachers, however, also stated that parents should facilitate practice at home, especially parents of the youngest students. Some of the teachers emphasized the importance of practicing a little bit every day, while others expressed that practicing should happen when the students have the desire to do it. Connected to this is also what should be the driving force for practicing. The teachers emphasized first and foremost a desire to play as the driving force, but also progression:

I believe it [the desire to practice] should come from the inside. So when I make rules for controlling it from the outside, it feels wrong. Because I believe that when the students get inspired, that force is much stronger than me telling them how much they should practice (North, teacher 2).

Related to what the students do at home between lessons is the contact and dialog between teachers and parents. About half of the teachers in the interview study reported that contact consists mostly of both parents and the teacher making contact if something requires it, although one of the teachers made contact with the parents on a weekly basis. The teachers accentuated the importance of dialog with parents. However, the teachers did not necessarily want the parents to be present in the lessons. Some of the teachers preferred that parents be present when the students are new and young, while others specified that they did not want parents to be present during the lessons. One teacher experienced that contact with parents was better after the digital lessons they were forced to have during the COVID-19 pandemic because of more meetings with the parents when “you [*via* the internet] show up in your students’ houses.”

In the survey, we asked the teachers to give two examples of music they used on their last day of teaching at a school of music and arts. One hundred and fifty teachers provided two examples each, and one teacher only one. Thus, we have 301 examples of teaching content. In addition to writing down the name and composer/artist of the music, the teachers also described how they worked with the specific repertoire. Second, we asked the teachers to give us one example of music they *prefer* to use as teaching content, and how they usually work with that content. One hundred and forty-seven teachers gave us one example each of their preferred repertoire. During the analysis, three main categories emerged from the material, namely (a) *educational aims*, (b) *working forms*, and (c) *focus areas*. Some of the categories also include subcategories, as shown in Table 1 below.

**Table 1:** Categories of *Ways of Working*, constructed through analyzing the data material from the teacher survey.

	Main categories	Subcategories
Ways of working	Educational aims	(a) Established musical goals
		(b) Open-ended musical goals
		(c) Students’ independence as goal
	Working forms	(a) Digital tools
		(b) Playing together
		(c) Representation of the music
	Focus areas	

#### 4.2.1. Educational aims

The category *educational aims* is divided into three subcategories: (a) *established musical goals*, where musical results are of importance, (b) *open-ended musical goals*, where the process is the main focus, and (c) *students’ independence as goal*, where neither musical results nor the musical process is most important, but rather the students’ musical independence. First, we present results from the 301 examples of teaching content from the last time of teaching, starting with the subcategory *established musical goals*. Here, the idea of “rehearsing” (“innstudere” in Norwegian) a musical piece is most prominent. Some of the teachers used that exact word,^5^ while others wrote that they “worked with the song piece by piece,” “worked with learning the parts,” or “worked through the repertoire slowly in order to help the students to learn it.” A commonality is that there is “something” that needs to be rehearsed; there is a musical piece already existing that the students ought to learn. “Imitation where the teacher demonstrates” and merely “teacher demonstration” were also reported by the teachers. There were also a few utterances pointing to an understanding of the repertoire as something that is pre-fixed and connected to the way it is supposed to sound. These utterances are “correcting mistakes the students make,” “practicing difficult parts,” and “working thoroughly and carefully with the piece.” Some of the teachers also wrote that they used the master-apprentice model.

The subcategory *open-ended musical goals* is a smaller category, with only a few of the teachers reporting to work with the repertoire in a way where the final musical product is not set from the beginning. These ways of working involve student engagement and creativity, focusing on the students’ own expression, creative abilities, and the process. It involves “improvisation,” “exploring the instrument,” “students making a variation of the piece,” and “making and/or working with accompaniment/chords.” “The students finding their own expression” was also reported by a few teachers. This could be understood as both a way of working in which the musical product is not pre-fixed, and also as a way of working in which the student’s independence is the goal. Within this final subcategory, *students’ independence as goal*, the teachers reported that they “guide the student into becoming her own teacher and being able to practice well at home,” and that they are “working on getting to know the piece and, together with the students, planning how to play it.”

Which music the teachers prefer to use as teaching content and how they usually work with it (150 examples), in many ways resemble how they work with the teaching content from the last time of teaching. Within the subcategory *established musical goals,* “rehearsing” (in Norwegian: “innstudere”) is also here most central, followed by “teacher demonstration,” “practicing difficult parts,” and using the master-apprentice model. In addition, a few of the teachers reported a focus on sight-reading. Within the subcategory *open-ended musical goals*, improvisation is more central here than it appeared in the teachers’ reports from their last day of teaching. “Making and/or working with accompaniment/chords” is also mentioned. Educational aims connected to the last subcategory, *students’ independence as goal*, are not frequently mentioned. Only a couple of the teachers reported to work with “students finding their own expression,” and one expressed that encouraging the students’ critical thinking about the repertoire and music in general is the main focus when working with musical pieces. There is, however, one thing that varies from how the teachers worked with music on their last day of teaching and which do not fit within any of the subcategories—namely, the idea of “working according to what the student needs.” This could be connected to the fact that the teachers did not necessarily report on what one student played at a specific time.

#### 4.2.2. Working forms

The category *working forms* is also divided into three subcategories: (a) *digital tools*, (b) *playing together*, and (c) *representation of the music*. We start with the results from the 301 examples of teaching content from the last time of teaching, focusing on *digital tools.* In more than one-third of the examples, the teachers reported the use of *digital tools* in one way or another. The ways they use them are primarily to listen to recordings, and secondly as play-alongs. Also mentioned were the use of YouTube, recording the lesson, finding sheet music or chords on the internet, and “learning by ear from recorded music.” In only two of the examples was a laptop reported as the actual instrument. *Playing together* is also a common working form in our material, with ensemble playing (playing with other students) as the main activity, followed by the teacher and student playing together, and the teacher accompanying the student. The most common *representation of the music* reported by the teachers were “using sheet music” and “working on reading the music,” followed by “using tablature” and “playing by ear.” A few reported the use of a combination of sheet music and playing by ear. How the teachers usually worked with their preferred teaching material essentially resembles how they reported working with it the last day of teaching.

#### 4.2.3. Focus areas

Connected to the last category, *focus areas,* the teachers reported focusing mostly on technique when working with the selected repertoire the last day of teaching. In addition, musical expression and dynamics, to play or sing the piece through, and rhythm and pulse, are also prominent in the data material. Some teachers focused on music theory, such as, for instance, analysis of chords and scales. Other areas of focus mentioned only by a few teachers were intonation, fingering, sound, and text, content, and interpretation. When asked how they usually work with their preferred repertoire, the teachers reported much as they did for their last day of teaching—namely, technique, musical expression and dynamics, rhythm and pulse, and music theory. What differs, though, is that focusing on text, content, and interpretation is more prominent.

## 5. Discussion

From the analysis of the survey and the interviews, we learn what music teachers in Norwegian schools of music and arts experience as meaningful approaches to selecting content and ways of working within instrumental music teaching. These can be summarized as: (a) the centrality of the students in the process of selecting content; (b) genre versatility, or that the students should be exposed to a broad range of musical genres and styles; and (c) the students being exposed to the “classical repertoire” within a genre or tradition, or its standard repertoire. The latter could be seen as part of a *material classical Bildung* tradition, where the goal is the acquisition of classical content within a culture, defined by what dominates and has status.

The centrality of the students is related to the importance of the students’ co-determination in the selection of teaching content, but also to the importance of the students being familiar with, and liking, the music that is taught. While this could be two sides of the same coin, the second could also imply that the teachers talk to the students and try out different musical pieces in order to find something the students enjoy playing. The idea of co-determination is even more explicit in the selection of content for the advanced students. The importance of co-determination could be seen in relation to the Scandinavian tradition of student involvement, where inclusion, individualization of teaching, and adaptive learning have for several years been prominent within educational contexts (Arnesen and Lundahl, 2006). Another finding from the analysis is that genres and musical styles within popular music are as common (actually, slightly more common) as classical music in Norwegian schools of music and arts. We suggest that there might be a connection between the focus on students’ co-determination and liking the music and popular music’s space in a school, which rests heavily on the long traditions of classical music (Karlsen and Nielsen, 2021) because more children are familiar with the popular music tradition than the classical. Other possible connections to the centrality of the students and the students’ independence are the emphasis on inner motivation for practicing, which several teachers spoke of, as well as the teachers’ utterances about parents not needing to be present in the lessons. The students’ co-determination is, however, emphasized more in the interviews and the survey question about preferred content than in the survey questions about content selection on the last day of teaching. From this, we can ask, does this imply that the teachers want to involve the students more in content selection than they actually do?

The emphasis on students’ co-determination and excitement for the music and its recognizability could be seen in relation to Klafki (2006, p. 129), who speaks of “the fruitful encounter between the children and the content,” which constitutes the purpose of instructional preparation. Co-determination and self-determination are central elements in Klafki’s (2011) critical-constructive Didaktik. The relevance of the teaching content for the students is also an important part of Klafki’s (2011) Bildung theory of *categorical Bildung.* Here, the content should be connected to the students’ world outside the school, both as a connection to the society as it is now and as significant for the students’ future. The teachers in our study coped with the latter issue in different ways: (a) by working toward the students’ independence and becoming their own teachers, (b) by teaching the standard repertoire within a genre so that the student will succeed if auditioning for higher music education; and (c) by presenting to the student a variety of genres and musical styles so that she has a broad ground on which to build her musical future. Combining these different approaches with content selection and ways of working could be difficult; it could also, however, be seen as a way to incorporate what Klafki named *exemplary teaching*; namely, the idea of enlightening abstract and fundamental principles by focusing on a few concrete examples that are connected to the students’ world outside the school (Johansen, 2007; Klafki, 2011; Straum, 2018). The importance of connecting to the world outside the school could also be seen in relation to Dyndahl and Ellefsen (2009), p. 9), who claim that the subject of music itself is “culturally contextual” and an arena for students’ identity constructions.

Although the students are central in the processes of choosing teaching content, findings from this study indicate that there are other areas of focus in the ways that teachers work with the content. This first and foremost concerns the centrality of established musical goals, both related to educational aims and working forms, among others, as most of the ways that teachers reported using digital tools are connected to established musical goals. This implies that acquisition of the content is central, which connects to the tradition of *material Bildung* (Klafki, 2011; Hanken and Johansen, 2021). It also relates to Nielsen’s (1998, 2007), central activity, *reproduction*, where performing and reproducing existing music is central. This does not have to conflict with emphasis on the student and her co-determination, but the idea of the student learning something that is “already there,” and working toward a musical goal set by others, could create tension toward students’ self-determination. Although the activity form *reproduction* is the most central one in our material, there are examples of teachers focusing on composing and improvising music in which the musical goals are open-ended. This connects to *formal Bildung* (Klafki, 2011; Hanken and Johansen, 2021). There are also a few examples in the data material of students’ independence as a goal, which could be understood within the *method-based formal Bildung* tradition, but which is also connected to Klafki’s (2011) critical-constructive Didaktik. The two activity forms of *interpretation* and *reflection* (Nielsen, 1998) are represented only to a limited extent in the data material.

## 6. Conclusion

Summing up, this study has demonstrated that various approaches to selecting content and ways of working with musical repertoire, as well as the use of diverse musical genres as teaching content, have been qualified as meaningful by the music teachers in the schools. As such, this exploration into what these music teachers construct as necessary didactical actions in their practice also show us what they view as legitimate ones produced by their *practical sense*. Further, although the teachers seem to some degree to be working by different logics of practice, the mere presence of such a variety of generated practices could to some extent be seen as contributing to making the schools of music and arts more accessible for a wider selection of people in terms of social class, musical preferences and so on, and thus facilitate cultural inclusion in a broader sense (see also Dyndahl et al. (2020) on the connection between musical genres and socio-cultural dynamics). What seems to be meaningful for the teachers in general is working “close to the student’s wishes and preferences,” but in ways which relate to a variety of Didaktik principles. In this way, one could say that the teachers understand their teaching in relation to the students’ world, vary their didactical strategies according to the students, and accommodate students’ negotiations and identifications. Thus, the teachers participate in constructing and reconstructing the “didactic identity” (Dyndahl and Ellefsen, 2009) of the music subject in schools of music and arts; they negotiate the teaching content according to their students and the cultural context in general.

## Data availability statement

The raw data supporting the conclusions of this article may be made available by the authors, under the reservation that the anonymity of the participants are upheld.

## Ethics statement

The studies involving human participants were reviewed and approved by NSD-Norwegian Centre for Research Data. The participants provided their written informed consent to participate in this study.

## Author contributions

The qualitative analyses were mainly carried out by AJ-L, but checked and discussed with all authors. All authors contributed equally to collecting the survey and interview data utilized in this publication.

## Funding

This work was supported by The Research Council of Norway (grant number 274936).

## Conflict of interest

The authors declare that the research was conducted in the absence of any commercial or financial relationships that could be construed as a potential conflict of interest.

## Publisher’s note

All claims expressed in this article are solely those of the authors and do not necessarily represent those of their affiliated organizations, or those of the publisher, the editors and the reviewers. Any product that may be evaluated in this article, or claim that may be made by its manufacturer, is not guaranteed or endorsed by the publisher.

## Footnotes

^1^Oslo is both a county and a municipality (and thus has one school of music and arts), and was therefore combined with Akershus when drawing municipalities for reasons of anonymity.

^2^Municipalities were classified as small (up to 9,999 inhabitants), medium-sized (10,000–74,999 inhabitants), or large (more than 75,000 inhabitants).

^3^Other genres included *American and Irish folk music*, *cross genre projects*, *Sami music*, *Scandinavian dance band music*, and *atonal/experimental music.*

^4^Kappleik is a competition in Norwegian instrumental and vocal folk music and village dance.

^5^The Norwegian equivalent of "innstudere."
